# Clinico-Demographic Parameters of Oral Fungal Infections: An Institutional Retrospective Study

**DOI:** 10.7759/cureus.55386

**Published:** 2024-03-02

**Authors:** Kamala S Devi, Abilasha R, Suvarna Kizhakkoottu

**Affiliations:** 1 Dentistry, Saveetha Dental College and Hospitals, Saveetha Institute of Medical and Technical Sciences, Saveetha University, Chennai, IND; 2 Oral Pathology and Microbiology, Saveetha Dental College and Hospitals, Saveetha Institute of Medical and Technical Sciences, Saveetha University, Chennai, IND

**Keywords:** systemic comorbidities, mucormycosis, denture stomatitis, angular cheilitis, candidiasis, deep fungal infections, superficial fungal infections, oral fungal infections, fungal infections

## Abstract

Introduction

Most fungal infections are responsive to antifungal therapy. However, failure to diagnose the same can significantly affect the quality of lives of patients. Timely identification of fungal infections and their association with varied demographic and clinical parameters will help in improving the prognosis of the patient. The present study aims to evaluate the prevalence of fungal infections among various age groups and genders and also to evaluate the association of fungal infections with demographic parameters.

Methods

This study included a sample size of n = 600. The demographic and clinical details were compiled and transferred to IBM SPSS Version 23 software (IBM Corp., Armonk, NY) for statistical analysis. Descriptive and Pearson chi-square tests were used to analyze the association of the type of fungal infection with gender, age, and comorbidities. A p-value of less than 0.05 is considered statistically significant.

Results

Angular cheilitis (40%, 240), followed by denture stomatitis (37.5%, 225), were the most common type of fungal infection among the sample population, and the elderly age group (51-72 years) was the most affected. Angular cheilitis was the most common infection among both males (21.4%, 128) and females (18.6%, 112), but candidiasis was reported more in females (18%, 108) than males (3%, 18) (p = 0.00). Angular cheilitis (32%, 192) and candidiasis (18%, 108) were more observed in association with anemia; however, denture stomatitis (34%, 204) was significantly higher among diabetics (p = 0.00).

Conclusion

The identification of associated systemic and demographic factors is as important as the treatment of fungal infection itself. The recognition of fungal infections and the role of parameters like age, gender, and systemic comorbidities in the development of fungal infections will have valuable implications for public health. Future research is required for a clear understanding of the same.

## Introduction

Fungal infections cause serious public health problems in society. In the era of COVID-19 and HIV infection, fungal infections associated with COVID-19 and HIV cases are life-threatening conditions that compromise the quality of life of a patient and can also lead to mortality [1]. The compromise in immunity associated with systemic corticosteroid treatment and uncontrolled diabetes mellitus are two risk factors for the development of fungal infections in individuals with HIV and COVID-19. Fungal infections can be superficial to systemic, mucosal, cutaneous, or subcutaneous type. *Candida*, *Aspergillus*, *Fusarium*, and *Mucorales* are a few fungal organisms that can cause opportunistic infections and healthcare-associated infections in patients with underlying diseases [2,3].

Fungal infections can be transmitted by inhalation of spores, percutaneous inoculation, and penetration into the mucosa by commensal organisms from contaminated food and drink and can affect internal organs [4]. Invasive fungal infections are generally uncommon but are devastating when they develop as a disease in immunocompromised patients. These infections are opportunistic; they occur when organisms to which the patient is frequently exposed gain entry to the body due to a reduction in host defenses [5]. Severe fungal infections are responsive to antifungal therapy, but they significantly affect the quality of lives of patients [6]. Some fungal species like blastomycosis, coccidioidomycosis, histoplasmosis, talaromycosis, paracoccidioidomycosis, and sporotrichosis can cause endemic infections in specific geographical regions.

In general, fungal infections can be superficial infections and invasive fungal infections. Superficial fungal infections incorporate those brought about by dermatophytes like fungus capitis, fungus faciei, fungus corporis, fungus unguium, fungus manuum, and non-dermatophytes like pityriasis versicolor, cutaneous candidiasis, fungus nigra, black piedra, and white piedra [7]. Fungus capitis is the most common shallow parasitic contamination among younger-grade students [8]. The most well-known and broadly dispersed superficial fungal organism is *Trichophyton rubrum* [9]. Dermatophytosis, pityriasis versicolor, and *Candida* can create keratinase, which permits them to utilize and live on human keratin-like skin, hair, and nails. Although dermatophytosis doesn't deliver mortality, it causes bleakness and represents a significant general medical issue, particularly in tropical nations like India [10,11]. Invasive fungal infections lead to fatal conditions with high rates of morbidity and mortality [12]. Organisms like *Candida*, *Aspergillus*, *Cryptococcus*, and *Pneumocystis* can cause systemic fungal infections [13].

In the oral cavity, both superficial and deep fungal infections are identified. Oral fungal infections are generally neglected unless they are severe, and oral fungal infections can be an indicator of underlying unrecognized systemic conditions. Since we are living in a COVID-19 era, deep fungal infections like mucormycosis and aspergillosis of the head and neck region of patients with systemic comorbidities and in elder age groups have become a common clinical presentation. The necessity to analyze the prevalence of fungal infections in the head and neck region in the present decade is valid. The present study aimed to evaluate the prevalence of fungal infections in different age groups and genders and also to find out whether the type of fungal infection has any association with age, gender, and comorbidities. Our team has extensive knowledge and research experience, which has translated into high-quality publications [14,15].

## Materials and methods

The present retrospective study was conducted after getting approval from the Institutional Ethical Committee in July 2020. The institutional ethical committee (Saveetha Institutional Human Ethical Committee) number of the study was SDC/SIHEC/2020/DIASDATA/0619-0320. For sample selection, we screened the data sheets of all the patients reported to the outpatient department of Saveetha Dental College, Saveetha Institute of Medical and Technical Sciences, Chennai, India, during the time of 2019-2022. Two independent observers were assigned to screen the three-year patient data and filtered patients reported with oral fungal infections. The filtered case sheets of oral fungal infections were further analyzed in detail by the same observers. The sample population selected for the present study included confirmed cases of oral fungal infections. Duplicate reports, cases with doubtful diagnoses, cases lacking follow-up data, and super-added fungal infections seen in carcinoma cases were excluded from the present study. After applying the exclusion criteria, the sample size of the present study was n = 600, which included two groups: females (n = 320) and males (n = 280). The demographic and clinicopathological details of the cases were retracted from the clinical database reserve of the institution. Demographic parameters like age and gender were included. Clinicopathological details like type of fungal infections and systemic comorbidities were also collected and extracted into Microsoft Excel 2016 (Microsoft® Corp., Redmond, WA) sheets. Age was further categorized into subgroups (18-30, 31-50, and 51-72 years) because these subgroups represent the population as young, middle-aged, and elderly. The collected demographic and clinical details were further reviewed by two independent observers. This was done to eliminate the repetition of cases, reduce discrepancy, and check the inclusion and exclusion criteria. Reviewed data were then transferred to IBM SPSS Version 23 software (IBM Corp., Armonk, NY) for statistical analysis. Descriptive analysis (frequency and percentage) was used to evaluate the prevalence of gender distribution, age group, and type of fungal infections. Pearson chi-square test was used to analyze the association of fungal infections with gender, age groups, and comorbidities. A p-value of less than 0.05 is considered statistically significant.

## Results

**Figure 1:**
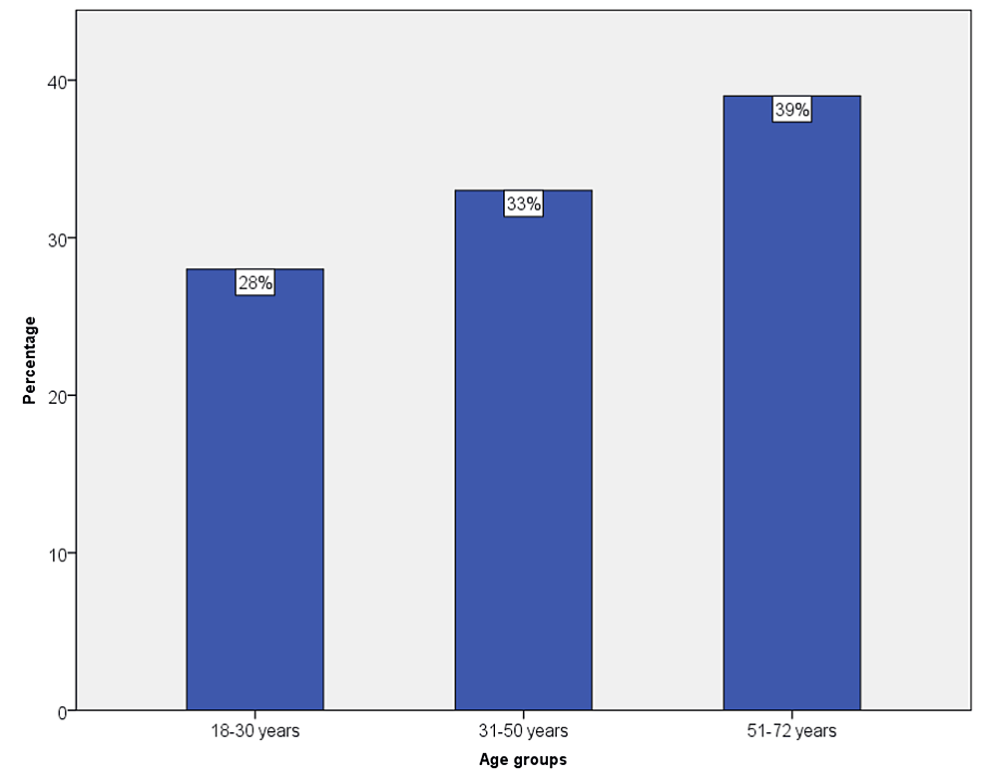
The percentage of distribution of the study population into different age groups such as 18-30, 31-50, and 51-72 years; 51-72 years (39%) were found to be more frequently affected with fungal infections followed by 31-50 years (33%), and the least affected were 18-30 years (28%).

All the cases included in the present study were not histopathologically confirmed, apart from a few mucormycosis cases. Since the current study does not deal with histopathological features but concentrates more on associations of fungal infections with comorbid conditions, age, and gender, we have included cases that were clinically diagnosed, responsive to antifungal therapy, and have a follow-up history. In the present study, the sample population included n = 600, of which females constituted about 53.68% (322), and males were 46.32% (278). The sample population was divided into groups according to age 18-30 (168), 31-50 (198), and 51-72 years (234) (Figure 1).

**Figure 2:**
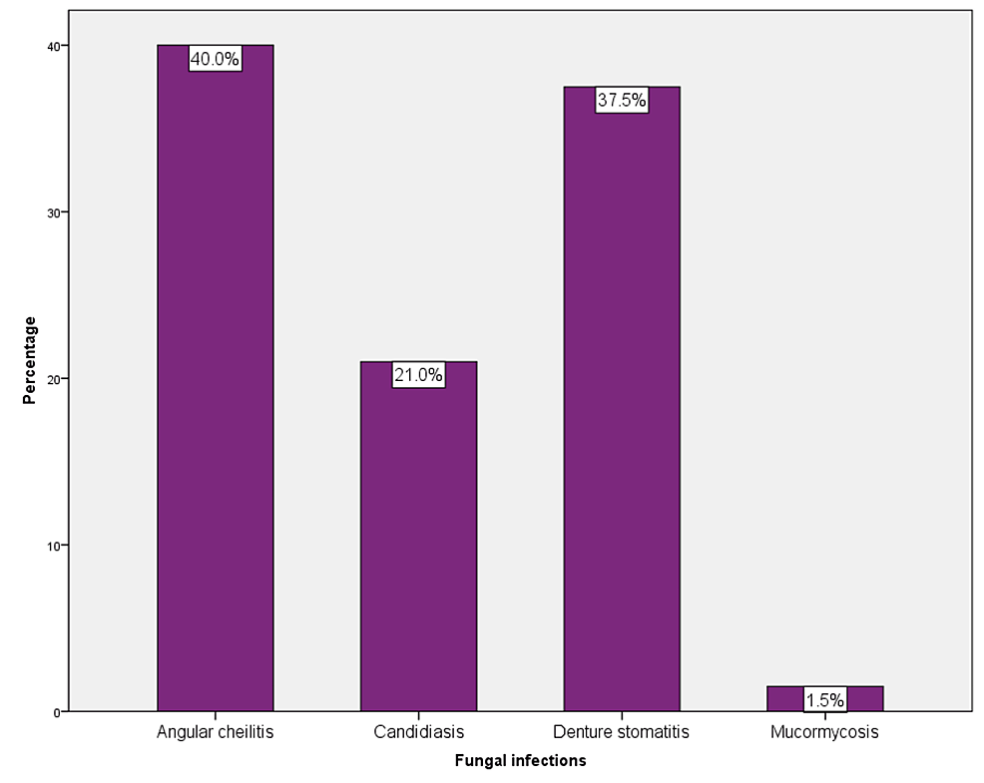
The percentage of various fungal infections in the sample population. Angular cheilitis (41%) was the most common, followed by denture stomatitis (37.5%) and candidiasis (21%). The least common was mucormycosis (1.5%).

Age groups 51-72 years (39%) were more affected by fungal infections, and 18-30 years (28%) were least affected. When the type of fungal infection was evaluated, angular cheilitis (40%, 246) was the most common, followed by denture stomatitis (37.5%, 225), candidiasis (21%, 126), and the least common was mucormycosis (1.5%, 9) (Figure 2). 

**Figure 3:**
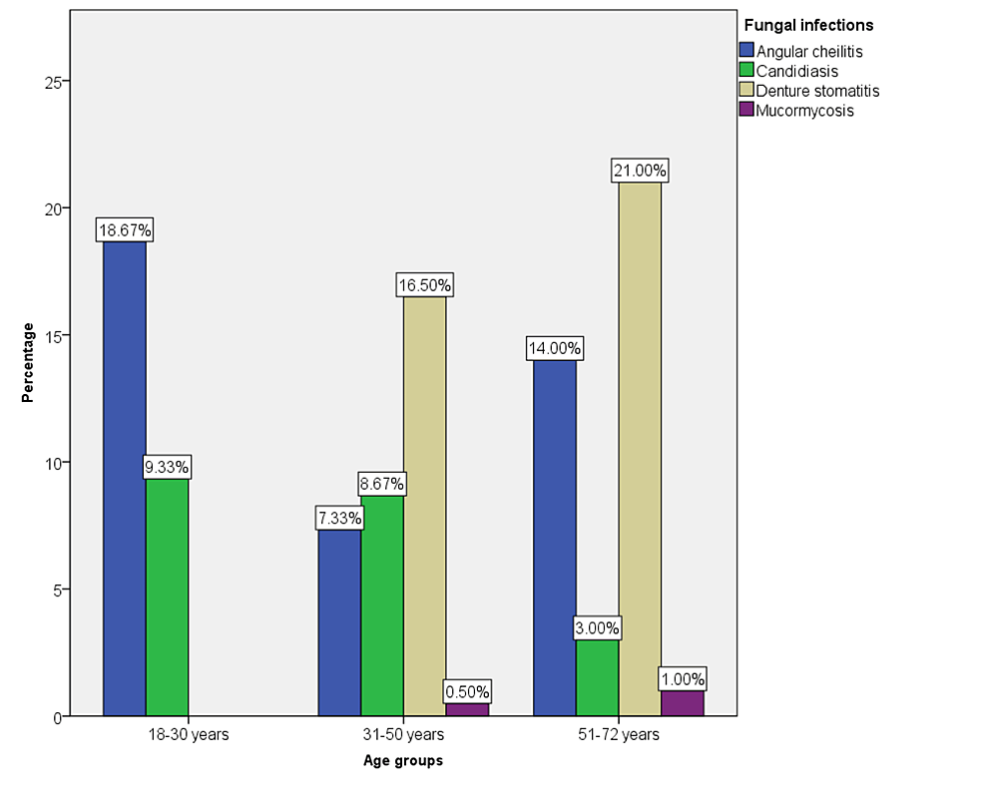
The association between age and type of fungal infections among the sample population. Among the 18-30 and 31-50 age groups, angular cheilitis was the most common, followed by denture stomatitis and candidiasis. However, in 51-72 years, denture stomatitis was the most common, followed by angular cheilitis and candidiasis. Mucormycosis was only seen in the 31-50 and 51-72 age groups (p = 0.00).

The association between fungal infections and age group was also found to be statistically significant, with a p-value of 0.00 (Figure 3).

**Figure 4:**
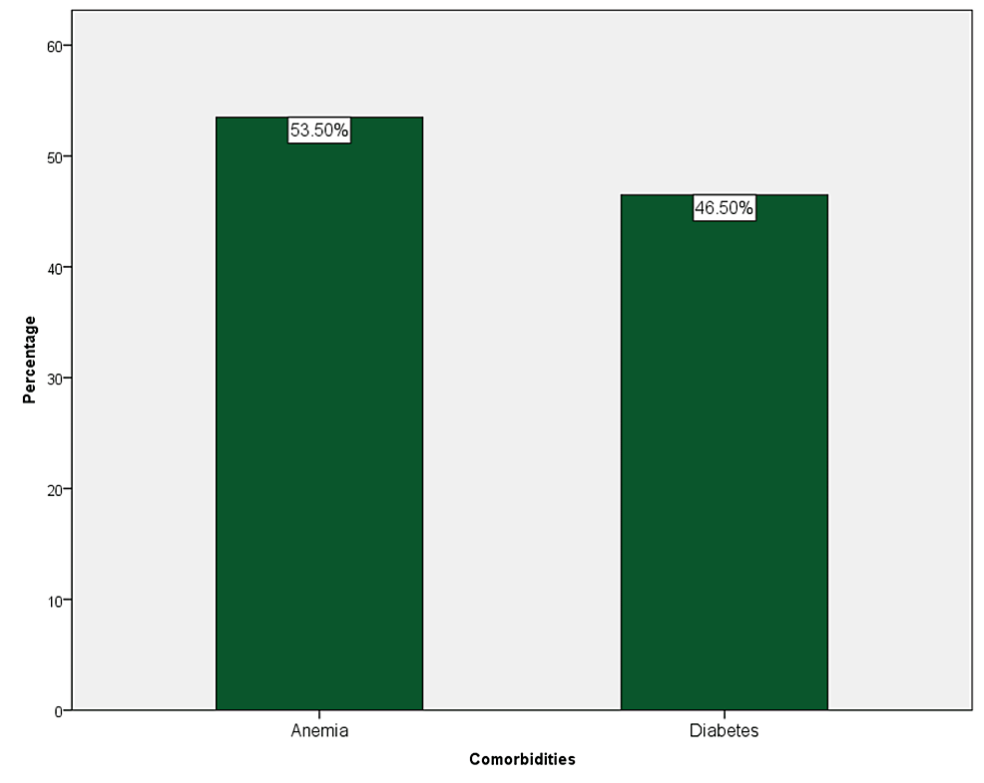
The comorbidities presented in patients with fungal infections. Anemia was more common and seen in 53.5% of the population, followed by diabetes mellitus and hypertension.

The study population was evaluated for existing comorbidities in association with fungal infections; 53.5% (321) of the study population were anemic, and 46.5% (279) were diagnosed with diabetes (Figure 4).

**Figure 5:**
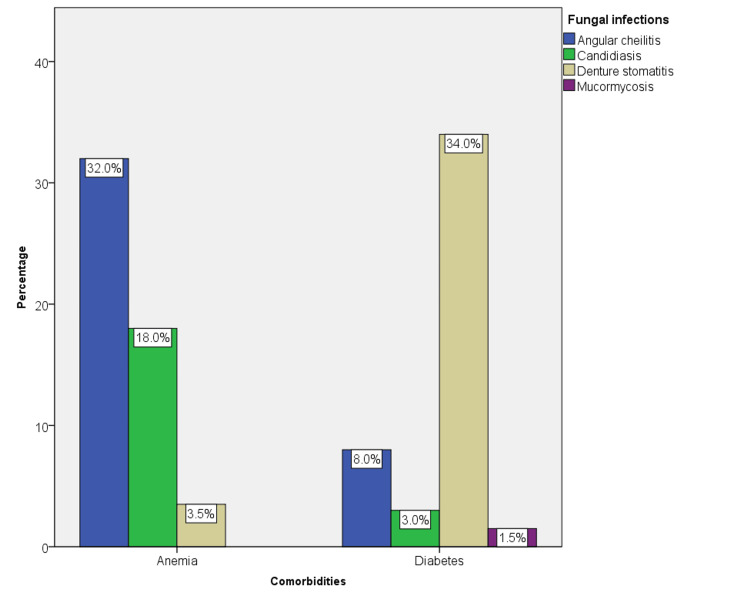
The prevalence of fungal infections among comorbid conditions such as diabetes and anemia. Among anemic patients, angular cheilitis was the most common fungal infection; however, denture stomatitis was found to be more common among diabetic patients (p = 0.00).

Angular cheilitis (32%) and candidiasis (18%) were more observed in association with anemia; however, denture stomatitis (34%) was significantly higher among diabetics (p = 0.00) (Figure 5).

**Figure 6:**
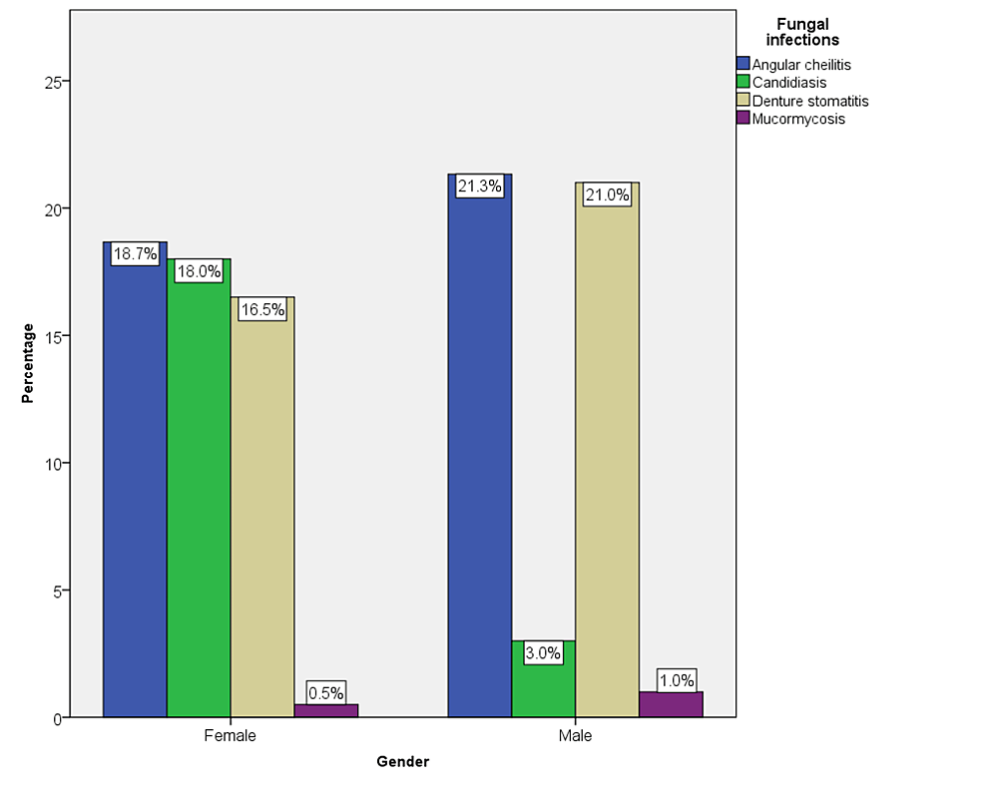
The prevalence of different fungal infections among males and females. Among males and females, angular cheilitis was the most common fungal infection. However, the prevalence of candidiasis was significantly higher in females compared to males. Mucormycosis was the least common fungal infection among both males and females (p = 0.00).

The association between the type of fungal infections and gender was also found to be statistically significant, with a p-value of 0.00. Females (18%) were more commonly affected with candidiasis compared to men (3%). Angular cheilitis (21.4%), denture stomatitis (21%), and mucormycosis (1%) were more commonly seen in males compared to females (18.6%, 18%, 0.5%) (Figure 6).

Tables 1-2 show the percentage and number of cases among different fungal infections in each age group and gender.

**Table 1:** The number and percentage of cases affected with different types of fungal infections among different age groups of the sample

Age groups	Angular cheilitis	Candidiasis	Denture stomatitis	Mucormycosis	Number of cases
18-30 years	81 (13.5%)	24 (4%)	63 (10.5%)	-	168 (28%)
31-50 years	87 (14.5%)	42 (7%)	63 (10.5%)	6 (1%)	198 (33%)
51-72 years	72 (12%)	60 (10%)	99 (16.5%)	3 (0.5%)	234 (39%)
Number of cases	240 (40%)	126 (21%)	225 (37.5%)	9 (1.5%)	600

**Table 2:** The number and percentage of cases affected with different types of fungal infections among males and females

Gender	Angular cheilitis	Candidiasis	Denture stomatitis	Mucormycosis	Number of cases
Male	128 (21.4%)	18 (3%)	126 (21%)	6 (1%)	278 (46.32%)
Female	112 (18.6%)	108 (18%)	99 (16.5%)	3 (0.5%)	322 (53.68%)

The variables cross-tabulated using Pearson chi-square analysis and the respective p-values are shown in Table 3.

**Table 3:** The evaluated variables and the Pearson chi-square and p-values of the cross-tabulation

Variables		Pearson chi-square (p-value)
Type of fungal infection	Comorbidities	0.00
	Age groups	0.00
	Gender	0.00

## Discussion

Most of the fungi are generally nonvirulent unless they come into contact with an immunocompromised patient, where the immunocompromised body defense allows them to infiltrate and grow. Only a few harmful fungi are aggressive and virulent enough to infect a healthy host [16].

Various studies have compared the gender prevalence of fungal infections in different populations; however, the majority of the studies are from the United States [17,18]. The results of the present study showed an increase in the prevalence of fungal infections in the female population (53.68%) compared to males (46.32%). This was per the previous literature [19,20]. Few studies have also discussed the influence of estrogen in the development of fungal infections, mainly *Candida albicans*, by promoting immune evasion [21].

Older adults are more susceptible to nosocomial infections, and as age advances, variations are observed in the host defense modulation [22]. Aging causes the immune system to lose the ability to protect against infections. This observation was in accordance with our present study, where the most commonly affected age group was found to be 51-72 years, and the least commonly affected were 18-30 years. In the younger age group, the immune system is well regulated and will rarely be affected by nosocomial and opportunistic infections [23].

Among the population studied, angular cheilitis was the most common fungal infection, followed by denture stomatitis and candidiasis, and the least common was mucormycosis. Even though mucormycosis was present only among 1.5% of the population studied, this was a major post-COVID-19 infection case. Previous research has reported that iron deficiency causes calcium overload and increased intracellular accumulation of reactive oxygen species (ROS), thereby leading to disturbed ion homeostasis and finally suppressing fungal growth [24]. However, aplastic anemia patients were proven to have an increased prevalence of fungal infections [25]. However, the present study result was contradictory to this observation since 53.5% of our study population had a history of anemia. Since anemia is a broad term, the non-categorization of anemia into subtypes can be a reasonable explanation for the opposing results. Despite this, 46.5% of the population had a history of diabetes mellitus, and this was following the existing literature. Since fungi feed on sugars, high blood sugar levels provide optimum energy for fungal organisms to thrive in the human body [26].

While studying the association between comorbidities and type of fungal infections, it was found that angular cheilitis was more common in anemic patients; however, denture stomatitis was more common among diabetes patients. This association was found to be statistically significant, with a p-value of 0.00. The etiology of angular cheilitis is multifactorial, which includes mechanical factors, infectious factors, nutritional deficiencies, or inflammatory dermatological conditions [27]. Mechanical factors include loss of vertical dimension, pooling of saliva, and improper orthodontic treatment and trauma. Habitual etiology is chronic use of tobacco products, smoking, chronic sun damage, and nutritional factors, including vitamin B2 deficiency [28]. In the present study, we have not addressed the etiology of angular cheilitis and have not considered whether the patients have any tobacco habits; hence, this can be a confounding factor in this study.

In the present study, denture stomatitis was associated with diabetes. A recent review and meta-analysis reported that patients with diabetes were more likely to develop denture stomatitis than non-diabetic denture wearers. Low pH and oxygen levels at the oral mucosal-prosthesis contact are linked to decreased salivary flow, and these elements most likely encourage *C. albicans* colonization and the eventual development of denture stomatitis [28]. Mucormycosis was seen only in association with diabetes mellitus. This could be due to the multi-system involvement of diabetes and Mucorales' need for iron for growth. Chronic and uncontrolled diabetes patients are more susceptible to mucormycosis due to the availability of free iron and acidic pH [29].

When the prevalence of fungal infections in each age group was studied, mucormycosis and denture stomatitis were found to be limited to older age groups. Aging upregulates immune system impairment, increases the prevalence of systemic diseases, increases the prevalence of COVID-19 infection, and results in edentualism leading to the use of dentures. COVID-19 virus-induced hypoxemia, steroid treatment-induced hyperglycemia, diabetic-associated high glucose levels and ketoacidosis, decreased phagocytic activity of WBC resulting in immune suppression, and elevated iron levels in blood make it easier for fungal growth. Hence, mucormycosis and denture stomatitis were mainly seen in older age group. Angular cheilitis was more prevalent in younger populations, and the reason for this could be attributed to vitamin B complex deficiency. The association was found to be statistically significant, with a p-value of 0.00

The association of gender and type of fungal infections was statistically significant with p-value of 0.00. Even though there were slight differences in the presentation of angular cheilitis and denture stomatitis among males and females, the variation in the prevalence of *C. albicans* was notable. The virulence of *C. albicans* in vivo is promoted by estrogen-dependent inactivation of the alternative complement system [20]. These findings imply that women would be more vulnerable to systemic *Candida* infections. The present study was in line with the abovementioned observation.

The main treatment modality implicated in fungal infections is anti-fungal agents (topical and systemic), and in invasive cases, anti-mycotic drug therapy must be complemented by surgery, especially in cases of mucormycosis or *Candida* abscesses [30]. Since the majority of invasive fungal infections are location-specific, preventive measures related to compromised immune responses play a significant role.

Limitations

Even though the present study evaluated the association of fungal infection and associated demographic parameters, this study was geographically and duration limited and also predominantly included the South Indian population. Hence, the limitations of the study were restricted geographic area coverage, a smaller sample population, and a lack of incorporation of etiological and histopathological confirmation. Incomplete exploration of environmental factors that may contribute to fungal infections, such as climate, occupational exposure, and living conditions, can also act as confounding factors. Considering a larger population from varied geographical areas at a multi-institutional level will help to arrive at a more precise conclusion.

## Conclusions

The recognition of the role of parameters like age, gender, and systemic comorbidities in the development of fungal infections and utilizing this for public health awareness can help in reducing the incidence and mortality rate from these infectious diseases. Oral fungal infections were more prevalent among the elderly population, and associated comorbid conditions have an important role in the development of specific fungal infections. Angular cheilitis was the most common fungal infection, and candidiasis was prevalent in the female population. Mucormycosis was reported in the elderly population and was associated with comorbidities. However, further research is needed, with the inclusion of samples from multiple geographic areas at a multi-institutional level. Incorporating the etiological factors of each disease and obtaining histopathological confirmation will provide a more precise conclusion regarding the association of varied clinico-demographic parameters with oral fungal infections.
